# DoE-Based Design of a Simple but Efficient Preparation Method for a Non-Effervescent Gastro-Retentive Floating Tablet Containing Metformin HCl

**DOI:** 10.3390/pharmaceutics13081225

**Published:** 2021-08-08

**Authors:** Byungsuk Kim, Youngjoo Byun, Eun Hee Lee

**Affiliations:** College of Pharmacy, Korea University, 2511 Sejong-ro, Sejong 339700, Korea; kbs528@korea.ac.kr (B.K.); yjbyun1@korea.ac.kr (Y.B.)

**Keywords:** metformin HCl, non-effervescent floating tablets (non-EFTs), sustained release (SR), spray-drying, low density solid dispersion, design of experiments (DoE), similarity factor (f_2_), bootstrap methodology

## Abstract

A sustained-release non-effervescent floating matrix tablet was prepared using a simple and efficient direct compression of spray-dried granules containing metformin hydrochloride and cetyl alcohol with hydroxypropyl methylcellulose K15M (HPMC K15M). The design of experiments was employed to explore the optimal composition of the tablet. The similarity factor was employed to evaluate the equivalence in dissolution profiles between the test tablets and Glucophage XR as a reference. Bootstrap analysis was used to eliminate the formulations for which the dissolution profile was potentially inequivalent to that of the reference. The optimized tablet consisting of 150 mg of cetyl alcohol and 17% HPMC K15M showed a dissolution profile comparable with that of the reference with a similarity factor of 52.41, exhibited a floating lag time of less than 3 s in buffer media, remained floating for 24 h, and reduced the tablet weight by about 20% compared to that of the reference. The current study sheds light on the potential use of non-effervescent gastro-retentive extended-release tablets for high-dose drugs using a simple and efficient direct compression method, and as a potential alternative treatment for Glucophage XR. This study also highlights the importance of a systematic approach to formulation optimization and the evaluation of the dissolution profile.

## 1. Introduction

Metformin hydrochloride (Met HCl) is an antidiabetic agent used to treat patients with type 2 diabetes. Unlike insulin and sulfonylureas, Met HCl does not promote weight gain and has thus been employed as an oral hypoglycemic agent for the first-line treatment of type 2 diabetes. It has also been shown to have a variety of other potential applications, including the treatment of obesity, HIV, gestational diabetes, cancer, and neurological abnormalities, in addition to its use with type 1 diabetes patients to reduce insulin resistance [1].

The pharmacokinetics of Met HCl can be represented by a two-compartment open model in which the plasma elimination half-life of 2–6 h corresponds to rapid elimination from the central compartment (i.e., beta half-life), while the terminal elimination half-life of 8–20 h corresponds to slow elimination from the deep compartment (i.e., gamma half-life) [2,3]. Because of the narrow absorption window in the proximal small intestine where the gastrointestinal absorption process ends, it was originally administered at high-unit doses (500 mg or 1000 mg) two to three times a day to achieve a glucose-lowering effect. However, the development of a gastro-retentive extended-release Met HCl tablet (Glucophage XR) offers a once-daily dosing option for Met HCl with a higher bioavailability and superior efficacy than immediate release Met HCl, thus improving patient compliance [4]. There have been significant efforts to reduce the size of Glucophage XR in order to improve patient compliance. Patients of all ages, especially elderly patients, have difficulty swallowing large tablets, which often leads them to stop taking their medication [5].

In recent decades, a number of studies have been conducted to develop new pharmacologically viable and therapeutically effective oral drug delivery systems. Gastro-retentive drug delivery systems have recently attracted significant attention as a strategy to produce high local concentrations in the stomach and to overcome bioavailability limitations related to plasma fluctuations, low solubility at an alkaline pH, narrow absorption windows, a short half-life, poor absorption in the low gastrointestinal tract, and low stability at an alkaline pH [6,7,8,9,10,11,12,13,14,15,16,17,18,19,20,21]. Various technologies, including floating, sinking, expandable, bioadhesive, raft-forming, super-porous hydrogel, and ion-exchange resin systems, have been introduced as potential gastro-retentive drug delivery systems. These techniques require specific excipients with special properties associated with the dosage form, such as a low density for non-effervescent floating systems, effervescence for effervescent floating systems, a high density for sinking systems, adhesiveness for bioadhesive systems, and expandability and swelling for floating, expandable, and raft-forming systems. The addition of excipients with sustained-release properties is also necessary for gastro-retentive systems.

The first floating system described by Davis in 1968 maintained buoyancy without gastric emptying because its density was lower than that of the gastric fluid. Based on this buoyancy mechanism, non- effervescent and effervescent systems have since been used to develop floating tablets. Effervescent floating tablets (EFTs) can be designed as a matrix using swellable polymers such as methylcellulose and chitosan and effervescent compounds such as sodium bicarbonate, tartaric acid, and citric acid. The carbon dioxide liberated from the matrix when it comes into contact with the gastric fluid due to the low pH in the stomach is trapped in the swollen hydrocolloid, thus increasing the buoyancy of the dosage form [22]. However, this system is influenced by the pH of the gastric acid and food in the stomach. In addition, EFTs exhibit a lag time between first contact with the gastric fluid and the point at which it starts to float.

Non-effervescent floating tablets (non-EFTs) are generally more buoyant than EFTs. Non-EFTs float without the liberation of carbon dioxide and are thus not affected by the pH of the stomach. Instead, the system swells when it comes into contact with the gastric fluid, leading to the entrapment of air in the gelatinous mass. The buoyancy of the matrix arises from this air entrapment process, which produces a bulk density that is lower than 1 [23,24]. Generally, both non-EFTs and EFTs improve patient compliance and bioavailability and reduce the dose frequency and risk of dose dumping. However, non-EFTs that lack the initial burst of carbon dioxide produced by EFTs may not be easily applied to high unit-dose drugs such as Met HCl. In this study, we attempted to develop high unit-dose Met HCl as a non-effervescent tablet with a reduced total tablet weight compared to the reference drug Glucophage XR, which has a total weight of 1040 mg.

Most floating tablets used for the delivery of Met HCl are EFTs (Table 1). 

Effervescence is produced by the addition of sodium bicarbonate with or without citric acid or potassium bicarbonate, and the ability to expand is provided by the use of hydroxypropyl methylcellulose (HPMC), sodium alginate, κ-carrageenan, PEO WSR 303, acacia gum, tarmarind seed gum, sodium CMC, sodium starch glycolate, or carbopol 934P. Met HCl EFTs are prepared using traditional tableting methods such as direct compression, wet granulation, and melt granulation. In contrast, non-EFTs require special preparation methods such as microsphere or bead formation, molding, and sublimation [39,40,41,42]. In the present study, we prepared non-EFTs using the simple and efficient direct compression of Met HCl granules prepared via spray-drying. Cetyl alcohol and HPMC K15M were used as the floating agent and extended-release agent, respectively [43].

Spray-drying is a single-step process that generates dried powder from a solution, suspension, or emulsion. In addition to its simplicity, spray-drying is robust in that it can ensure consistent powder properties throughout the process and represents a continuous and easily scalable process [44]. Most powder particles produced via spray-drying are spherical, with a narrow particle size distribution. The properties and size of the powder particles can also be modified by adjusting feed solution properties such as the viscosity, chemical stability and composition, and process parameters such as the inlet/outlet temperature, feed rate, nozzle type, and airflow pattern [45]. Due to the intrinsic characteristics of spray-drying, it can generate low-density granules, which is a prerequisite for non-EFTs.

The quality by design (QbD) approach is regarded as necessary to assure the quality of pharmaceutical products. QbD begins by defining the quality target product profile, understanding the process and the material attributes, and controlling the process based on sound science and quality risk assessment. One of the tools for QbD is the design of experiments (DoE). In the response surface methodology (RSM), the optimal approach to DoE, randomization, is used to minimize the effects of uncontrolled variables and possible disturbances, thus eliminating random error. Furthermore, all planned design points are used to determine the correlation between the characteristics of individual materials and product quality, as well as that between the interaction of more than two materials and product quality, with statistical data analysis providing an accurate interpretation of the results [46]. Based on these results, a design space is established and a robust formulation that guarantees product quality can be successfully determined. The present study used a two-level full factorial design (FFD) and the RSM from the DoE. By designing the optimal composition using these design approaches, a formulation that led to non-effervescent floating and sustained release that had a dissolution profile similar to that of the original Glucophage XR, and that had minimal weight, was successfully developed. The similarity factor was used to compare the dissolution profile of the newly developed non-EFT and Glucophage XR, and the prediction accuracy of models for the drug release rate was confirmed using bootstrap methodology with the R program.

## 2. Materials and Methods

### 2.1. Materials

Met HCl was purchased from Harman Finochem, Ltd. (Aurangabad, India). Sodium alginate was purchased from FMC BioPolymer (Protanal^®^ LF 5/60, Philadelphia, PA, USA). Povidone K-30 was purchased from BASF (Kollidon^®^ 30, Ludwigshafen, Germany). Polyethylene glycol 6000 (PEG 6000) was supplied by Sanyo Chemical Industries Ltd. (Kyoto, Japan). Polyvinyl alcohol was purchased from Merck KGaA (Darmstadt, Germany). HPMC K15M was purchased from Colorcon Asia Pacific Pte. Ltd. (METHOCEL^®^ K15M, Merchant Square, Singapore). Cetyl alcohol was purchased from Pilipinas Kao, Inc. (KALCOL 6870P, Misamis Oriental, Philippines). Magnesium stearate was purchased from Peter Greven GmbH & Co. KG (Bad Münstereifel, Germany). Glucophage XR was purchased from Merck Serono Ltd. (Feltham, UK). Methanol and ethanol were of high-performance liquid chromatography (HPLC) analytical grade.

### 2.2. Methods

#### 2.2.1. Preparation of a Solid Dispersion of Met HCl with Excipients

First, 500 mg of Met HCl and 250 mg of each of the excipients listed in Table 2 were dissolved in 750 mL of water and 750 mL of 70% ethanol (*v*/*v*). A solid dispersion (SD) was produced by spray-drying [47] the clear solution using a Mini Spray Dryer B-290 (Büchi Labortechnik AG, Flawil, Switzerland) coupled with a 0.7 mm two-fluid nozzle and nitrogen gas [48]. The process parameters included an inlet temperature of 110–120 °C, an outlet air temperature of 55–60 °C, a spray flow at a rate of 50 mL/min, an aspiration rate of 65%, and a feed rate of 9 mL/min. The spray-dried SDs were collected in a cyclone (Büchi Labortechnik AG, Flawil, Switzerland) and stored at 25–31 °C and a relative humidity of 23–30% in a desiccator containing silica gel until further analysis.

#### 2.2.2. Gas Chromatography (GC)

The spray-dried SD was tested with a residual solvent (ethanol) using an Agilent 7890A gas chromatograph (Agilent, CA, USA) equipped with a flame ionization detector connected to an Agilent G1888 headspace sampler. GC analysis was performed on a capillary DB-624 column (0.32 mm × 30 mm, 1.8 um; Agilent, Santa Clara, CA, USA). The initial separation temperature within the column was 40 °C, which was raised to 80 °C at a heating rate of 10 °C/min at 100 kPa with a retention time of 5 min and an injection volume of 1000 uL. The carrier gas was helium with a flow rate of 1 mL/min.

#### 2.2.3. Scanning Electron Microscopy (SEM)

The morphology of each SD particle consisting of Met HCl and the excipients was analyzed with a scanning electron microscope (SEM) [49] using a ZEISS SIGMA 500 SEM (ZEISS, Oberkochen, Germany). Samples were prepared for analysis by placing SD powder on conductive carbon tape fixed to an aluminum mount. The samples were sputter-coated for 50 s using a Hitachi Ion Sputter E-1030 (Hitachi Science Systems, Ltd., Chiba, Japan) at 20 mA and 0.07 Torr (N) with a thin layer of gold-palladium (Au-Pd) prior to SEM analysis. Under high vacuum mode, a secondary electron (SE) detector was used with a beam voltage of 3.0–5.0 kV. Data were collected and analyzed using SmartSEM Touch Ver. 5.09 (ZEISS, Oberkochen, Germany) [50].

#### 2.2.4. Powder X-ray Diffraction

The powder patterns for Met HCl, spray-dried Met HCl, and the SDs were obtained using a D8 ADVANCE with DAVINCI (Bruker AXS Inc., GmbH, Karlsruhe, Germany) with Cu Kα radiation and equipped with a high-speed LynxEye detector. Samples were analyzed over a 2θ range of 4–40° with increments of 0.02° at a rate of 6°/min. The data were analyzed using DIFFRACplus Eva (Bruker AXS Inc., GmbH, Karlsruhe, Germany) [49].

#### 2.2.5. Bulk Density

Bulk density was measured by filling approximately 10 g (*m*) of the test sample into a 100-mL graduated cylinder without compacting. After reading the apparent volume (*V*_0_) to the nearest graduated unit (mL), the bulk density in g/mL was calculated using the formula *m*/*V*_0_.

#### 2.2.6. Particle Size Distribution

Particle size distribution analysis was performed using a laser diffraction particle size analyzer (Mastersizer 2000, Malvern Panalytical, Malvern, UK) equipped with a dry powder feeder (Scirocco 2000, Malvern Panalytical, Malvern, UK). The air pressure, feed rate, and measurement time were set at 2 bar, 60%, and 10 s, respectively. The median particle diameter (d0.5) was determined based on 3.0 g samples.

#### 2.2.7. Tablet Preparation

The SDs produced using the spray dryer were mixed with HPMC K15M and magnesium stearate in a 3 L polyvinyl bag for 5 min. The mixtures were allowed to stand for 1 h and then weighed and fed manually using only one station of the punch die mounted on a Riva PICCOLA rotary tablet press (RIVA, Shropshire, UK). An oval punch with a width and length of 19 mm x 10 mm was used. The tableting pressure was 1200 to 1400 Nwt, and the hardness of the tablet was 3 kp to 6 kp. The hardness of the tablets was measured using a hardness tester (PTB 311, Pharma Test, Hainburg, Germany).

#### 2.2.8. Floating Tests

In vitro floating assessment was conducted by modifying the method described by Rosa et al. [51]. The prepared tablets were loaded into Nessler tubes containing a 0.1 M HCl buffer (pH 1.2). The floating ability of the tablets was evaluated via visual observation for 24 h.

#### 2.2.9. In Vitro Dissolution Analysis

The dissolution profile of 500 mg of Met HCl from the non-EFTs (12 units/test) was evaluated using a USP Dissolution Apparatus II (708-SD, Agilent, Santa Clara, CA, USA). The dissolution test was carried out in 900 mL of a 0.1 N HCl buffer (pH 1.2) containing 0.03 M NaCl at 37 ± 0.5 °C at a paddle rotation rate of 50 rpm in consideration of the sustained release characteristics of the tablet. After adding one tablet to each dissolution basket, a 10 mL aliquot was withdrawn from each dissolution basket after 15, 30, 45, 60, 120, 240, 480, 720, 1080 and 1440 min, and the same amount of fresh medium was replaced each time to maintain the sink conditions.

#### 2.2.10. Release Kinetics Model

The in vitro release of Met was modeled using zero-order kinetics, first-order kinetics, the Higuchi model, and the Korsmeyer–Peppas model in order to better understand the drug release mechanisms. R^2^ was used to determine the best-fitting model. The values of *n* were calculated for the Korsmeyer-Peppas model. The relationship between the released Met and the matrix erosion of non-EFTs was determined.

#### 2.2.11. High-Performance Liquid Chromatography (HPLC)

The amount of Met in each aliquot during the dissolution analysis was determined using HPLC (Agilent, CA, US) equipped with UV/Visible spectroscopy at 255 nm. The mobile phase consisted of a 0.2 M KH_2_PO_4_ buffer (pH 5.6), methanol, and acetonitrile (60:20:20, v/v/v), with a flow rate of 1.0 mL/min. A Kromasil C18 column (4.6 × 150 mm, 5 μm; Nouryon, Amsterdam, Netherlands) was used, and the injection volume was 10 μL.

#### 2.2.12. Design of Experiments (DoE)

An experimental design of screening and optimization steps was chosen as the statistical approach for the analysis of the tablet formulation as the critical factor and the dissolution profile as a response. Design-Expert^®^ 9 (Stat-Ease, Inc., Minneapolis, MN, USA) was employed to identify the design space and optimal conditions for the associated factors. The optimal cetyl alcohol/HPMC K15M ratio was explored using DoE. The range was set at 50–250 mg for cetyl alcohol and 5–25% for HPMC K15M, which represented 611.0–1071.3 mg per tablet based on an average weight of 1040 mg ± 3% (*n* = 10) for Glucophage XR tablets (Met 500 mg, with 5% Mgst included). The two-level full factorial design (FFD), a cost-effective prescreening design, is presented in Table S1 [52,53]. It was employed as a screening step to confirm the effect of (A)-cetyl alcohol and (B)-HPMC K15M and the interaction between (A)-cetyl alcohol and (B)-HPMC K15M as the X variables on the response (R)-mean dissolution profile as the Y variable. These Y variables were used to obtain the similarity factor for sustained-release formulations based on the EP and USP. The three Y variables were coded as R1–R3. The time points used to compare the dissolution profile for the test tablet to that for Glucophage XR as a reference were at 60, 240 and 480 min, corresponding to drug release rates of 30, 60, and 80%, respectively. With the ranges obtained from the screening process, formulation optimization was conducted using the response surface model (RSM) (Table S2) [46].

#### 2.2.13. Dissolution Profile Comparison

The similarity factor (f_2_) introduced by Moore and Flanner (1996) was calculated to compare the difference in the mean dissolution of the test tablets and the reference at the set time points Equation (1). The expected value of f_2_ was calculated using Equation (2) as an asymptotically unbiased estimate E(f_2_). The 90% confidence interval (CI) estimated using the bootstrap methodology (bootf2BCA_v1.1 in the R environment, Copyright © 2016–2018, Aleksander Mendyk) was used to calculate the percentile CI (PI). For the cutoff similarity of 50, the lower limit of the PI and the bias-corrected and accelerated CI (Bcα CI) presented by Efron were employed [54].

(1) $$f_{2}=50\times \log\left\{{\left[1+\frac{1}{P}\sum_{i=1}^{P}{\left(\mu_{\mathrm{ti}}-\mu_{\mathrm{ri}}\right)}^{2}\right]}^{-0.5}\times 100\right\}$$

where P is the number of observations considered, μ_ti_ and μ_ri_ are the mean dissolution at the *i*-th time point for the test and reference products, respectively.

(2) $$E\left(f_{2}\right)=50\times \log\left\{{\left[1+\frac{1}{P}\sum_{i=1}^{P}{\left(\chi_{\mathrm{ti}}-\chi_{\mathrm{ri}}\right)}^{2}+\sum_{i=1}^{P}\left(s_{\mathrm{ti}}^{2}+s_{\mathrm{ri}}^{2}\right)/n\right]}^{-0.5}\times 100\right\}$$

where χ_ti_ and χ_ri_ are the mean dissolution of the 12 tablets measured at the *i*-th time point for the test and reference products, respectively, and s_ti_^2^ and s_ri_^2^ are the sample variance at *i*-th time point for the test and reference products, respectively.

#### 2.2.14. Swelling and Matrix Erosion

The swelling of optimized non-EFTs was evaluated by measuring their weight [55]. The experiments were conducted in the USP Dissolution Apparatus II. The initial non-EFT weight was precisely weighed using a Mettler-Toledo ML-203 electronic balance. The weighed tablet was placed in a dissolution basket, which was then dipped in a dissolution vessel containing 900 mL of 0.1 M HCl buffer (pH 1.2) at 37 ± 0.5 °C. The tablet was collected at constant time intervals and lightly blotted with filter paper to remove the excess water, and the swollen tablet was weighed. The rate of increase in the tablet weight, which can be attributed to the adsorption or absorption of water, was calculated using Equation (3) at each time point. After the swelling experiment, the swollen tablet was dried in a convection oven at 40 °C for 12 h [56]. The dried tablets were cooled at room temperature and weighed. The matrix erosion was calculated at each time point as a percentage using Equation (4).

(3) $$\mathrm{Swelling}\;=\frac{\mathrm{weight}\;\mathrm{of}\;\mathrm{the}\;\mathrm{swollen}\;\mathrm{tablet}\;-\mathrm{initial}\;\mathrm{weight}\;\mathrm{of}\;\mathrm{the}\;\mathrm{tablet}}{\mathrm{initial}\;\mathrm{weight}\;\mathrm{of}\;\mathrm{the}\;\mathrm{tablet}}\times 100\%$$

(4) $$\mathrm{Matrix}\;\mathrm{erosion}\;=\frac{\mathrm{initial}\;\mathrm{weight}\;\mathrm{of}\;\mathrm{the}\;\mathrm{tablet}\;-\mathrm{weight}\;\mathrm{of}\;\mathrm{the}\;\mathrm{dried}\;\mathrm{tablet}}{\mathrm{initial}\;\mathrm{weight}\;\mathrm{of}\;\mathrm{the}\;\mathrm{tablet}}\times 100\%$$

## 3. Results and Discussion

### 3.1. Screening of Excipients for Co-Spray-Dried Solid Dispersion of Met HCl

SDs of Met HCl were prepared by co-spray-drying Met HCl with the excipients sodium alginate, PVP K-30, PEG6000, polyvinyl alcohol (PVA), HPMC K15M, and cetyl alcohol [43,57,58,59]. Excipient screening was conducted to select an excipient that was suitable for floating and direct compression. The weight of Glucophage XR containing 500 mg of Met HCl was about 1040 mg ± 3%. Considering the amount of Met HCl, the amount of the excipient for the preparation of the SDs was initially set at 250 mg.

**Figure 1:**
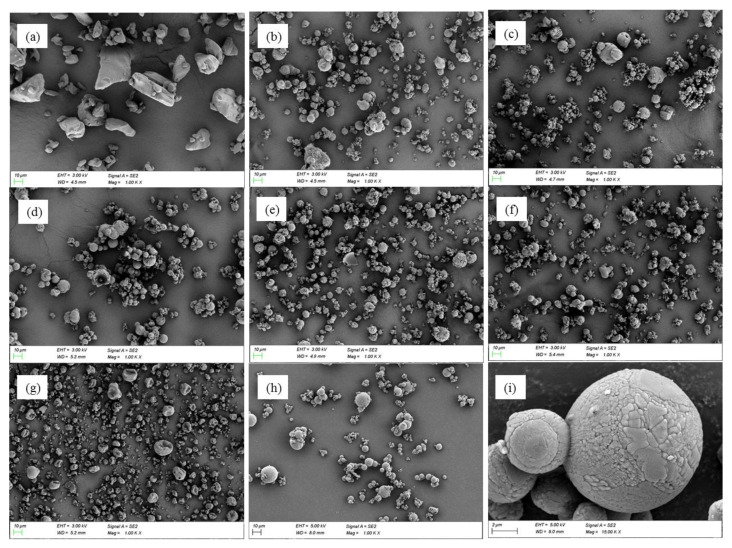
SEM of pure Metformin HCl and co-spray dried solid dispersion with the polymer. (**a**) Pure Met HCl, (**b**) pure Met HCl SD, (**c**) SD with sodium alginate, (**d**) SD with PVP K-30, (**e**) SD with PEG6000, (**f**) SD with polyvinyl alcohol (PVA), (**g**) SD with HPMC K15M, (**h**) SD with cetyl alcohol (×1000), (**i**) SD with cetyl alcohol (×15,000).

Figure 1 presents SEM images of pure Met HCl, spray-dried Met HCl, and co-spray-dried SDs. The SDs were spheroidal particles when prepared using the two-fluid nozzle of the spray dryer, while the pure Met HCl crystals were large and prismatic [60]. It is well-known that large prismatic Met HCl has poor compressibility. However, the direct compression of Met HCl was feasible in the form of spherical SDs with cetyl alcohol (Figure 1). The powder patterns of pure Met HCl, Met HCl SDs, co-spray-dried SDs, and Glucophage XR showed that Met HCl was present in a crystalline form (Figure S1).

Table 3 shows the particle size distribution, the Met content in the SDs, the bulk density of the spray-dried SDs, and the residual solvent of ethanol. When the mean droplet size was set to 5–20 μm, the particle size distribution of SDs had a range of 5.06–7.62 μm (d0.5). The particle size distribution of the SDs containing cetyl alcohol was 7.15 μm (d0.5). The bulk density of the SDs containing water-soluble polymers was 0.40–0.51 g/cm^3^, while that of the SDs containing cetyl alcohol (a fatty alcohol) was the lowest of the tested excipients (0.3 g/cm^3^). The SDs were mixed with a lubricant (magnesium stearate, Mgst) to produce a total weight of 757.58 mg per tablet, and these tablets were placed in 0.1 N HCl buffer (pH 1.2) to evaluate their floating properties. Cetyl alcohol was the only excipient that floated in the buffer, possibly due to its low density. The residual solvent for SD6 using 70% ethanol was 134.1 ppm, which satisfied the criteria of less than 5000 ppm specified by USP < 467 > related to residual solvents for ethanol (Class 3) using GC.

### 3.2. Design of Experiments (DoE) for the Non-Effervescent Floating Sustained-Release Formulation

**Figure 2:**
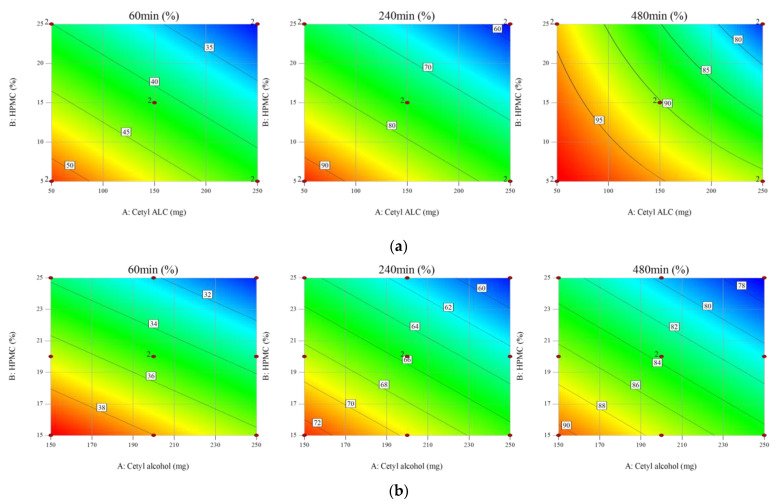
Contour plots of (**a**) the two-level full factorial design (FFD) and (**b**) the response surface method (RSM).

During the screening process, cetyl alcohol was selected as the floating agent. The ratio between cetyl alcohol and HPMC K15M was thus a critical factor for the non-effervescent floating sustained release of Met HCl. The two-level FFD presented in Table S1 was used as a screening step to confirm the effect of (A)-cetyl alcohol and (B)-HPMC K15M and the interaction between (A)-cetyl alcohol and (B)-HPMC K15M as X variables on the response (R)-mean dissolution profile as the Y variable. The time points used to compare the dissolution profile for the test tablets to that for the reference were 60, 240, and 480 min, corresponding to drug release rates of 30, 60, and 80%, respectively. The experiment had a 2 × 2 design with two replicates, one block, and two center points per block. The experimental order was randomized. Cetyl alcohol and HPMC K15M had *p*-values of <0.05 for all three drug release rates, which indicates that they were critical factors affecting the dissolution profile. The contour plots in Figure 2a show that the drug release rate decreased as the cetyl alcohol and HPMC K15M increased. The coefficients of determination of R^2^ 93.42 (Adj. R^2^ 91.54) for 60 min, R^2^ 99.20 (Adj. R^2^ 98.97) for 240 min, and R^2^ 98.42 (Adj. R^2^ 97.63) for 480 min indicate a strong correlation between the X and Y variables.

**Figure 3:**
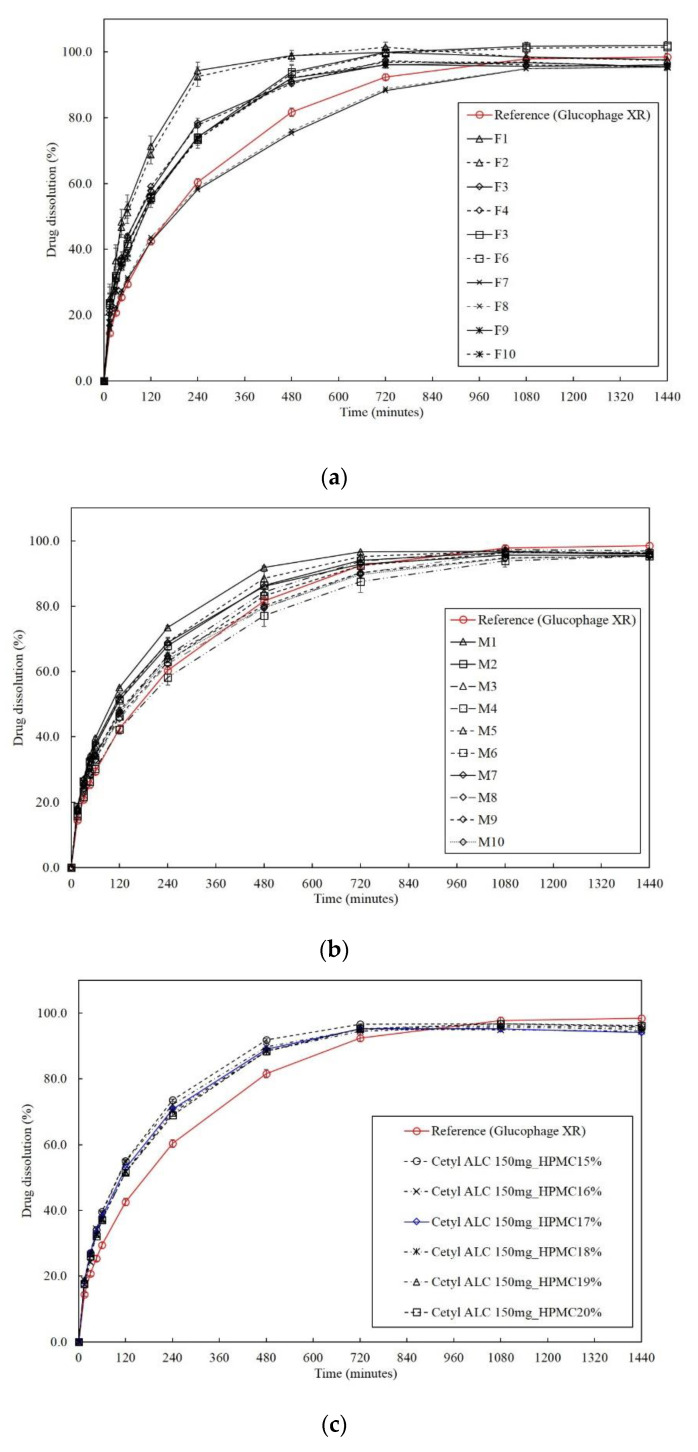
(**a**) Dissolution profiles for the two-level full factorial design (FFD); (**b**) dissolution profiles for the response surface method; (**c**) dissolution profiles for the optimized formulations including the dissolution profile of Glucophage XR as the reference product.

The difference of dissolution profiles between the test tablet and the reference was compared using the f_2_ (mean ± SD, *n* = 12) (Figure 3a). The f_2_ calculated using Equation (1) ranged from 29.35 to 45.77 for 50 mg of cetyl alcohol and from 47.26 to 48.50 for 150 mg of cetyl alcohol. On the other hand, it ranged from 69.38 to 71.46 when 250 mg of cetyl alcohol and 25% of HPMC K15M were used (Table S1).

The tablets containing 50 mg of cetyl alcohol (F1 and F2 in Table S1) did not float, while those containing 150 or 250 mg of cetyl alcohol floated immediately and remained floating for 24 h in dissolution vessels containing 0.1 N HCl buffer. Based on the floating test, the formulation containing 50 mg of cetyl alcohol was excluded from the RSM.

The RSM was used to optimize the formulation because more precise statistical analysis is possible by repeating the center point and performing the experiment [46]. In the RSM, the amount of cetyl alcohol was set at either 150 or 250 mg and HPMC K15M at 15 or 25%. In the central composite face, the axial point was placed at the center of the surface of the cube to generate reasonable stability of the predicted variance, and it was sufficient to perform one or two center point experiments [61]. As shown in Table S2, a total of 10 experimental points consisting of two repetitions of the center point and eight noncentral points were designed, and the mean dissolution profiles of the test tablets at 60, 240, and 480 min (mean ± SD, *n* = 12) were compared with that of the reference (Figure 3b). The f_2_ was also calculated, and the floating lag time and the floating retention time were evaluated. Except for M1, which contained 150 mg of cetyl alcohol and 15% HPMC K15M, all of the formulations from M2 to M10 exhibited dissolution profiles similar to the reference and met the f_2_ requirement. All the formulations from M1 to M10 showed a floating lag time of less than 3 s and a floating retention time of more than 24 h.

The *p*-value of the regression model at each time point was obtained using analysis of variance (ANOVA), and was less than 0.05, confirming the validity of the regression equation models used in the RSM (Table 4). Similar to the FFD, a negative correlation between the X and Y variables was observed (Figure 2b). At 480 min, a quadratic equation was recommended for FFD, while a linear equation model was used for the RSM with a *p*-value of 0.0081. A quadratic equation model was recommended for (R3) 480 min with a *p*-value of 0.0047 and an Adj. R^2^ of 90.96% for A*A (cetyl alcohol*cetyl alcohol). However, because the *p*-value for (A)-cetyl alcohol was 0.028 (*p* < 0.05), a linear equation model was used in the analysis, excluding A*A, and the Adj. R^2^ was 67.49%.

The choice of the linear equation model for (R3) 480 min was validated by comparing the accuracy of the results predicted using each equation model with external validation.

### 3.3. Prediction Using an External Validation Set

The prediction accuracy of the models (actual equations) reported in Table 4 was validated by comparing the experimentally obtained and the predicted dissolution profiles using either a linear or a quadratic equation derived from RSM analysis. The external validation set (E1–E6) consisted of the dissolution profiles of the formulations containing 150–250 mg cetyl alcohol and 5–10% HPMC K15M, which were not included in the RSM analysis.

The evaluation method used Equation (5) with a 95% CI. The root mean squared error of prediction (RMSEP), which is determined by the distribution of the prediction errors, was calculated and used as a simple criterion for the predictive ability of the model [62,63].

(5) $$\mathrm{RMSEP}=\sqrt{\left(1/n\right)\sum_{i=1}^{n}{\left(y_{i}-{\hat{y}}_{i}\right)}^{2}}$$

where *y_i_* is the reference value for the external validation set (*i* = 1,…,n), and *ŷ_i_* is the prediction for *y_i_*.

As shown in Table S3, the RMSEP for the linear equation model for (R3) 480 min was 0.66, and the RMSEP for the quadratic equation model was 2.19, confirming the higher accuracy of the linear equation model compared to the quadratic equation model. Therefore, the linear equation was selected for further analysis.

### 3.4. Optimization of the Formulation Using the RSM

Formulation optimization was conducted using the RSM. Our goal was to obtain low-weight non-EFTs, so we chose a range between 15% (M1) and 20% (M5) for HPMC K15M with 150 mg of cetyl alcohol.

Using the models (actual equations) determined using the RSM in Table 4, the optimal formulation that satisfies the cutoff value of >50 should contain more than 15.98% HPMC K15M (Table S4). However, the experimentally obtained f_2_ when 16% HPMC K15M (P1) was included was 49.85. When 17% HPMC K15M (P2) was included in the formulation, both the predicted f_2_ (51.68) and the experimentally obtained f_2_ (52.41) ensured the equivalence of the dissolution profiles (Figure 3c) between the optimized formulation and the reference (Table 5).

### 3.5. Release Kinetic Models

As shown in Table 5, the Met release profiles of non-EFTs were best described when the Korsmeyer-Peppas model was used. All formulations had an *n* value lower than 0.45, corresponding to the Fickian diffusion mechanism and indicating that diffusion was the primary drug release mechanism from the non-EFTs [64].

### 3.6. Evaluation of Dissolution Profile Equivalence via Bootstrap Analysis

The dissolution profile equivalence of the test tablets in comparison to the reference was evaluated by comparing the overall dissolution profile, the f_2_, and the dissolution values at every time point (Table S3). The f_2_ values calculated using Equation (1) were obtained experimentally and from the linear model derived from the RSM. Bootstrap analysis also generated the f_2_, the asymptotically unbiased estimate E(f_2_), the PI, and the Bcα CI. In the bootstrap analysis, the effect of using more than 500 replicates was negligible; thus 500 replicates were used to characterize the distribution of the f_2_ [65,66,67].

The bootstrap methodology applied in this study was also used as a criterion to select the formulation for a sustained-release non-EFT with minimal weight. The f_2_ of the sample mean, the f_2_ obtained from the linear model, and the f_2_, E(f_2_), PI, and Bcα CI from the bootstrap methodology are shown in Table 5. The predicted value for HPMC K15M was 16%. The f_2_ for the mean dissolution profile predicted by the linear model was 50.04 when 16% HPMC K15M was used in the formulation. However, the f_2_ for the mean dissolution profile for 150 mg of cetyl alcohol and 16% HPMC K15M (P1) obtained experimentally was 49.85, and the f_2_ and the E(f_2_) estimated using the bootstrap analysis were 49.86 and 49.81, respectively. In addition, the lower limit of the PI was 48.75 and the lower limit of the Bcα CI was 48.84, which were slightly below the cut-off value of 50 for the f_2_. However, the sample mean f2 for the formulation (P2) containing 150 mg of cetyl alcohol and 17% HPMC K15M was 52.41, and the bootstrap analysis result showed that the f_2_ was 52.41, the E(f_2_) was 52.43, the lower limit of the PI was 51.42, and the lower limit of the Bcα CI was 51.45. Bootstrap analysis also confirmed that the optimized formulation (P2) should contain at least 17% HPMC K15M. The tablet weight of the optimized formulation was 833.40 mg, which was about 20% less than the average weight of 1040 mg (± 3%, *n* = 10) of the reference (Glucophage XR). The floating lag time of the tablet was less than 3 s, and the floating retention time was more than 24 h.

In the case of the formulation (E6) containing 250 mg of cetyl alcohol and 10% HPMC K15M, the f_2_ for the mean dissolution profile was 50.70, the f_2_ for the mean dissolution profile predicted by the linear model was 51.60, and the f_2_ and the E(f_2_) estimated using the bootstrap analysis were 50.81 and 50.64, respectively. These results indicated the equivalence of the dissolution profiles for the test tablets and the reference. However, the lower limit of the PI was 49.41 and the lower limit of the Bcα CI was 49.43, which were slightly below the cut-off value of 50 for the f_2_. In conclusion, using the bootstrap methodology, the dissolution profiles could be compared and interpreted more accurately, and the limitations of the prediction model for the mean dissolution profile employed in this study could be improved.

### 3.7. Floating, Swelling, and Erosion Testing of the Sustained-Release Non-Effervescent Floating Tablet

**Figure 4:**
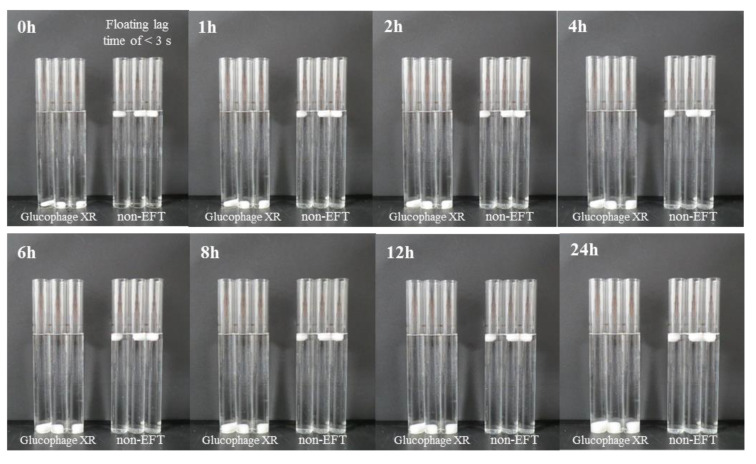
Comparison of the floating ability between Glucophage XR tablets (*n* = 3) and non-EFTs (*n* = 3).

As shown in Figure 4, when three Glucophage XR tablets and three tablets with the optimized formulation (P2: 150 mg of cetyl alcohol and 17% HPMC K15M) were each placed in a 100 mL Nessler tube, the tablets with the optimized formulation floated in the buffer media for 24 h with a floating lag time of <3 s (Figure 4 and Figure S2). Because Glucophage XR was not designed as a floating tablet, it sank in the buffer media and swelled due to HPMC K15M, which is also a well-known excipient for extended release.

Controlled-release gel-forming matrix tablets are known to undergo swelling and erosion during dissolution. A hydrophilic drug such as Met HCl penetrates and/or diffuses through the hydrated layer of HPMC K15M and is thus released in a controlled manner [68,69]. Erosion also plays an important role in releasing a drug, especially hydrophobic drugs, during dissolution [70].

**Figure 5:**
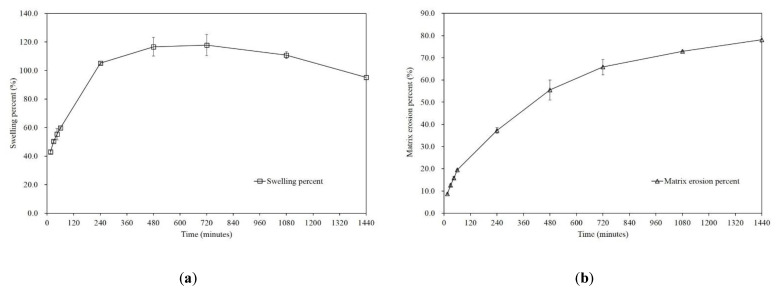
Percentage of (a) swelling and (b) matrix erosion for tablets with the optimized formulations (mean ± SD, *n* = 3).

We analyzed the change in the swelling (Equation (3), Figure S3a) and matrix erosion (Equation (4), Figure S3b) of the tablets with the optimized formulation. Initially, the tablet was 17.6 mm wide, 8.8 mm long and 8.3 mm thick, and the hardness of the tablet ranged from 29.4 N (3 kp) to 58.8 N (6 kp). It was clearly shown that rapid swelling occurred at the initial stage of dissolution, and the progress of swelling seemed to slow down after 6 to 24 h (Figure 5a). Erosion progressed linearly during the entire process (Figure 5b). This behavior further confirms the drug release model finding that Met HCl is released via a diffusion-controlled mechanism.

It was also noted that the tablets maintained their framework during the entire dissolution process, while the tablets were eroded after removing water. This characteristic can be a solution for the discharge of the floating tablet via the pylorus when in a lying position after taking a tablet. Because the pylorus is about 2–3 mm in diameter during digestion, dilating to about 12.8 ± 7.0 mm in diameter during the interdigestive phase, floating swollen tablets may avoid being passed through the pyloric sphincter [71,72]. In addition, matrix erosion behavior can indicate that the tablets can be easily removed after completing gastro-retentive and extended-release purposes [73].

## 4. Conclusions

In this study, sustained-release non-EFTs were prepared using a simple but efficient direct compression of co-spray-dried granules of Met HCl and cetyl alcohol with HPMC K15M. The granules prepared using spray-drying with cetyl alcohol had a low density, thus exhibiting good floating ability without the help of an effervescent agent, while also demonstrating good compressibility due to their spherical shape. Cetyl alcohol appeared to play an important role in the floating behavior, but spray-drying was also a critical influence in this respect.

The design of experiments was employed to explore the optimal composition of the tablet. The similarity factor was employed to evaluate the equivalence in dissolution profiles between the test tablets and Glucophage XR as a reference. Bootstrap analysis was used to eliminate the formulations for which the dissolution profile was potentially inequivalent to that of the reference. RSM analysis indicated that the drug release rate decreased linearly as the cetyl alcohol and HPMC K15M content increased. The newly developed and optimized tablet (P2), consisting of 150 mg of cetyl alcohol and 17% HPMC K15M, exhibited a lag time of less than 3 s in buffer media, remained floating for 24 h, and had a tablet weight that was about 20% lower than that of the reference (Glucophage XR). Based on the f_2_ value of 52.41, we conclude that the dissolution profile of the proposed Met HCl tablet was also comparable with that of the reference. The PI produced using bootstrap analysis provided more conservative ranges for the f_2_, allowing the formulation on the borderline to be eliminated from consideration.

This study sheds light on the potential use of non-effervescent gastro-retentive extended-release tablets with a high-dose drug using a simple and efficient direct compression, offers a potential alternative treatment to Glucophage XR, and highlights the importance of a systematic approach to the optimization of formulations and the evaluation of dissolution profiles.

## Figures and Tables

**Table 1:** Summary of reported floating drug delivery systems for Metformin HCl.

NEFT/EFT	Preparation Methods	Polymers for Extended-Release Property	Excipients for Floating Property	Title
Effervescent	Wet granulation	HPMC K4M, HPMC K15M, HPMC K100M	SB	Gastro-floating bilayer tablets for the sustained release of metformin and immediate release of pioglitazone: preparation and in vitro/in vivo evaluation [25]
		Guar gum, κ-carrageenan, HPMC K100	SB, CA	Formulation and characterization of metformin hydrochloride floating tablets [26]
		HPMC K4M, carbopol 934P	SB	Formulation and evaluation of floating drug delivery system of metformin hydrochloride [27]
		PVP, TSG, HPMC	SB, CA	Influence of water-soluble polymers on the in vitro performance of floating mucoadhesive tablets containing metformin [28]
		HPMC, PEO, SSG	SB	Design and in-vitro evaluation of sustained release floating tablets of metformin HCl based on effervescence and swelling [29]
		HPMC	PB	Development and in vitro evaluation of sustained release floating matrix tablets of metformin hydrochloride [30]
	Melt-granulation	HPMC	SA, SB, CA	Optimization of a metformin effervescent floating tablet containing hydroxypropylmethylcellulose and stearic acid [31]
		HPMC K4M, HPMC K15M, HPMC K100M, AG	SB	Effervescent floating tablets of metformin HCl developed by melt granulation. Part I: effect of hydrophilic polymer on biopharmaceutical properties [32]
		HPMC K15M, HPMC K100M, AG	SB	Development of floating tablets of metformin HCl by thermoplastic granulation. Part II: In vitro evaluation of the combined effect of acacia gum/HPMC on Biopharmaceutical performances [33]
	Direct compression	HPMC K4M, HPMC K100M, SSG, PVP-K-30, MCC	SB, CA,	Formulation and evaluation of bilayered floating tablets of metformin hydrochloride [34]
		Sodium alginate, sodium CMC	SB	Gastroretentive drug delivery of metformin hydrochloride: formulation and in vitro evaluation using 3^2^ full factorial design [35]
		HPMC K15M, κ-carrageenan	SB	Application of simplex centroid design in formulation and optimization of floating matrix tablets of metformin [36]
		PEO WSR 303	SB	Effects of formulation and process variables on gastroretentive floating tablets with a high-dose soluble drug and experimental design approach [37]
	Polymer coating	Polyvinyl acetate, ammonio-methacrylate copolymer type A	SB, CA	Physiological relevant in vitro evaluation of polymer coats for gastroretentive floating tablets [38]
Non-effervescent	Mold–hollow-core floating tablet (HCFT)	HPMC K100M, MCC	n/a	Novel self-floating tablet for enhanced oral bioavailability of metformin based on cellulose [39]
	Wet-granulation, Sublimation	PEO WSR 301, HPC	D, L-Camphor	Preparation of highly porous gastroretentive metformin tablets using a sublimation method [40]
	Emulsion solvent evaporation method	n/a	Liquid paraffin, Span 60, petroleum ether	Pharmacokinetic and pharmacodynamics evaluation of floating microspheres of metformin hydrochloride [41]
	Beads	n/a	Gelucire 43/01	Development of Gelucire 43/01 beads of metformin hydrochloride for floating delivery [42]
	AG: acacia gum; CA: citric acid; HPC: hydroxypropyl cellulose; HPMC: hydroxypropylmethylcellulose; MCC: microcrystalline cellulose; PB: potassium bicarbonate; PEO: polyethylene glycol; PVP: polyvinylprrolidone; SA: stearic acid; SB: sodium bicarbonate; sodium CMC: sodium carboxymethylcellulose; SSG: sodium starch glycolate; TSG: tarmarind seed gum			

**Table 2:** The feasibility test of co-solid dispersion (SD) formulation for floating.

Components (mg)	Solvent/mL	SD1	SD2	SD3	SD4	SD5	SD6
Metformin HCl	-	500	500	500	500	500	500
Sodium alginate	Water/750	250	-	-	-	-	-
PVP K-30	Water/750	-	250	-	-	-	-
PEG 6000	Water/750	-	-	250	-	-	-
Polyvinyl alcohol	Water/750	-	-	-	250	-	-
HPMC K15M	Water/750	-	-	-	-	250	-
Cetyl alcohol	70% Ethanol/750	-	-	-	-	-	250
Magnesium stearate	-	7.58	7.58	7.58	7.58	7.58	7.58
Tablet total (mg)	-	757.58	757.58	757.58	757.58	757.58	757.58

**Table 3:** Results of the feasibility test for co-solid dispersion (SD) formulation.

Test (*n* = 3)	SD1	SD2	SD3	SD4	SD5	SD6
Bulk density of SD (g/cm^3^)	0.41 ± 0.01	0.41 ± 0.01	0.42 ± 0.01	0.40 ± 0.01	0.51 ± 0.01	0.30 ± 0.01
Particle size distribution of SD (d 0.5, um)	7.15 ± 0.23	5.32 ± 0.13	7.62 ± 0.22	6.38 ± 0.25	5.06 ± 0.15	7.15 ± 0.18
Content (%) of metformin in SD	99.31 ± 0.73	98.59 ± 0.68	99.30 ± 0.79	99.26 ± 0.59	99.67 ± 0.50	99.75 ± 0.56
Floating of tablet in 0.1 N HCl buffer (pH 1.2)	Not floated	Not floated	Not floated	Not floated	Not floated	Floated
Residual solvent of SD6 (Ethanol < 5000 ppm)	n/a	n/a	n/a	n/a	n/a	134.10 ± 2.08

**Table 4:** Analysis of variance (ANOVA) table of the response surface methodology for three time points.

ANOVA (*p*-Value < 0.05)	Y Variable (Response)		
	(R1) 60 min	(R2) 240 min	(R3) 480 min
Model	<0.0001	<0.0001	0.0081
A: Cetyl alcohol	0.0002	0.0002	0.0280
B: HPMC K15M	<0.0001	<0.0001	0.0086
R-Squared	96.50%	95.45%	74.71%
Adj R-Squared	95.38%	94.15%	67.49%
Coded equation	R1 = +35.06 − 1.72 × A − 2.95 × B	R2 = +65.61 − 3.05 × A − 4.17 × B	R3 = +83.64 − 2.98 × A − 3.90 × B
Actual equation	R1 = +53.73 − 0.03 × A − 0.59 × B	R2 = +94.48 − 0.06 × A − 0.83 × B	R3 = +111.17 − 0.06 × A − 0.78 × B

**Table 5:** Summary of tablet weights for all designed formulations, the fitting of the release kinetics models, and the results of bootstrap analysis (90% CI).

Cetyl alcohol (mg)	HPMC K15M (%)	Code	Mass (mg)	In Vitro Drug-Release Model					Sample Mean (f_2_)	Bootstrap Analysis (500)			
				Zero-order	Higuchi	Korsmeyer-Peppas				f_2_	E(f_2_)	PI	Bcα
				R^2^	R^2^	R^2^	n	k					
150	5	E1	722.2	0.6193	0.8050	0.9116	0.3531	0.9749	37.68	37.69	37.67	(36.88, 38.49)	(36.84, 38.44)
	10	E2	764.2	0.6621	0.8402	0.9284	0.3729	0.9111	42.98	42.98	42.95	(42.29, 43.62)	(42.35, 43.67)
	15	M1	812.4	0.6939	0.8648	0.9432	0.3743	0.8992	47.36	47.36	47.35	(46.61, 48.13)	(46.63, 48.15)
	16	P1	822.8	0.6921	0.7573	0.9399	0.3779	0.8819	49.85	49.86	49.81	(48.75, 51.00)	(48.84, 51.12)
	17	P2	833.4	0.7089	0.7847	0.9551	0.3664	0.9094	52.41	52.41	52.43	(51.42, 53.60)	(51.45, 53.65)
	20	M5	866.6	0.7355	0.8950	0.9619	0.3802	0.8716	55.41	55.44	55.43	(53.69, 57.29)	(53.62, 57.17)
	25	M3	928.5	0.7782	0.9233	0.9761	0.3939	0.8216	69.08	69.09	68.67	(65.58, 71.99)	(66.25, 72.91)
200	5	E3	777.8	0.6397	0.8221	0.9223	0.3488	0.9806	40.09	40.06	40.04	(39.47, 40.69)	(39.50, 40.74)
	10	E4	823.5	0.6923	0.8635	0.9437	0.3617	0.9305	47.68	47.73	47.64	(46.79, 48.53)	(46.91, 48.75)
	15	M7	874.9	0.7419	0.8995	0.9611	0.3677	0.9611	56.15	56.12	56.05	(54.44, 57.69)	(54.44, 57.69)
	20	M9	933.2	0.7892	0.9305	0.9779	0.3752	0.8638	69.32	69.08	69.02	(67.13, 70.80)	(67.24, 70.88)
	25	M8	1000.0	0.8109	0.9435	0.9837	0.3925	0.8127	77.88	77.86	77.50	(75.89, 79.16)	(76.54, 80.05)
250	5	E5	833.2	0.6594	0.8380	0.9318	0.3306	1.0235	42.40	42.45	42.43	(41.69, 43.18)	(41.70, 43.19)
	10	E6	882.3	0.7222	0.8856	0.9559	0.3600	0.9327	50.70	50.81	50.64	(49.41, 51.73)	(49.43, 51.79)
	15	M2	937.4	0.7496	0.9047	0.9677	0.3704	0.8940	58.15	58.13	57.99	(55.51, 60.81)	(55.48, 60.79)
	20	M6	1000.0	0.7856	0.9279	0.9772	0.4016	0.7939	76.76	76.58	76.43	(73.01, 79.94)	(72.82, 79.60)
	25	M4	1071.3	0.8249	0.9513	0.9862	0.4126	0.7412	75.47	75.62	75.17	(68.91, 82.46)	(69.42, 83.50)

## Data Availability

Data is contained within the article.

## Supplementary Materials

The following are available online at https://www.mdpi.com/article/10.3390/pharmaceutics13081225/s1, Table S1: The full factorial design for a screening step and dissolution profiles (mean ±SD, *n* = 12), and information on similarity factors, floating lag time and floating retention time of non-EFTs. Table S2: The response surface methodology for optimization step and dissolution profiles (mean ± SD, *n* = 12), and information on similarity factors, floating lag time and floating retention time of non-EFTs. Table S3: Comparison of dissolution profiles (mean ± SD, *n* = 12) for the external validation set, model prediction accuracy and bootstrap analysis. Table S4: Comparison of the dissolution profiles for the optimized formulations predicted using the RSM with the experimentally obtained dissolution profiles. Figure S1: PXRD patterns for crystalline metformin HCl, spray-dried solid dispersions, and Glucophage XR. Figure S2: Images of non-EFTs in the vessels after the 24-h dissolution test (*n* = 12). Figure S3: Images of non-EFTs during (a) swelling and (b) erosion tests over time. Non-EFTs were taken out from the vessels at predetermined times during the dissolution test.

## References

[1] Rojas L.B.A., Gomes M.B. (2013). Metformin: An old but still the best treatment for type 2 diabetes. Diabetol. Metab. Syndr..

[2] Setter S.M., Iltz J.L., Thams J., Campbell R.K. (2003). Metformin hydrochloride in the treatment of type 2 diabetes mellitus: A clinical review with a focus on dual therapy. Clin. Ther..

[3] Robert F., Fendri S., Hary L., Lacroix C., Andréjak M., Lalau J.D. (2003). Kinetics of plasma and erythrocyte metformin after acute administration in healthy subjects. Diabetes Metab..

[4] Stepensky D., Friedman M., Srour W., Raz I., Hoffman A. (2001). Preclinical evaluation of pharmacokinetic–pharmacodynamic rationale for oral CR metformin formulation. J. Control. Release.

[5] Christofides E.A. (2019). Practical insights into improving adherence to Metformin therapy in patients with type 2 diabetes. ADA.

[6] Lopes C.M., Bettencourt C., Rossi A., Buttini F., Barata P. (2016). Overview on gastroretentive drug delivery systems for improving drug bioavailability. Int. J. Pharm..

[7] Qin C., Wu M., Xu S., Wang X., Shi W., Dong Y., Yang L., He W., Han X., Yin L. (2018). Design and optimization of gastro-floating sustained-release tablet of pregabalin: In vitro and in vivo evaluation. Int. J. Pharm..

[8] Jain S.K., Jangdey M.S. (2008). Lectin conjugated gastroretentive multiparticulate delivery system of clarithromycin for the effective treatment of *Helicobacter pylori*. Mol. Pharm..

[9] Hardikar S., Bhosale A. (2018). Formulation and evaluation of gastro retentive tablets of clarithromycin prepared by using novel polymer blend. Bull. Fac. Pharm. Cairo Univ..

[10] Inukai K., Takiyama K., Noguchi S., Iwao Y., Itai S. (2017). Effect of gel formation on the dissolution behavior of clarithromycin tablets. Int. J. Pharm..

[11] Mostafavi A., Emami J., Varshosaz J., Davies N.M., Rezazadeh M. (2011). Development of a prolonged-release gastroretentive tablet formulation of ciprofloxacin hydrochloride: Pharmacokinetic characterization in healthy human volunteers. Int. J. Pharm..

[12] Chavanpatil M.D., Jain P., Chaudhari S., Shear R., Vavia P.R. (2006). Novel sustained release, swellable and bioadhesive gastroretentive drug delivery system for ofloxacin. Int. J. Pharm..

[13] Fu J., Yin H., Yu X., Xie C., Jiang H., Jin Y., Sheng F. (2018). Combination of 3D printing technologies and compressed tablets for preparation of riboflavin floating tablet-in-device (TiD) systems. Int. J. Pharm..

[14] Kagan L., Lapidot N., Afargan M., Kirmayer D., Moor E., Mardor Y., Friedman M., Hoffman A. (2006). Gastroretentive Accordion Pill: Enhancement of riboflavin bioavailability in humans. J. Control. Release..

[15] Hwang K.-M., Cho C.-H., Tung N.-T., Kim J.-Y., Rhee Y.-S., Park E.-S. (2017). Release kinetics of highly porous floating tablets containing cilostazol. Eur. J. Pharm. Biopharm..

[16] Kim S., Hwang K.-M., Park Y.S., Nguyen T.-T., Park E.-S. (2018). Preparation and evaluation of non-effervescent gastroretentive tablets containing pregabalin for once-daily administration and dose proportional pharmacokinetics. Int. J. Pharm..

[17] Ngwuluka N.C., Choonara Y.E., Kumar P., du Toit L.C., Modi G., Pillay V. (2015). An optimized gastroretentive nanosystem for the delivery of levodopa. Int. J. Pharm..

[18] Sarkar D., Nandi G., Changder A., Hudati P., Sarkar S., Ghosh L.K. (2017). Sustained release gastroretentive tablet of metformin hydrochloride based on poly (acrylic acid)-grafted-gellan. Int. J. Biol. Macromol..

[19] Patil S., Talele G.S. (2015). Gastroretentive mucoadhesive tablet of lafutidine for controlled release and enhanced bioavailability. Durg Deliv..

[20] Sawicki W. (2002). Pharmacokinetics of verapamil and norverapamil from controlled release floating pellets in humans. Eur. J. Pharm. Biopharm..

[21] Jiménez-Martínez I., Quirino-Barreda T., Villafuerte-Robles L. (2008). Sustained delivery of captopril from floating matrix tablets. Int. J. Pharm..

[22] Baumgartner S., Kristl J., Vrečer F., Vodopivec P., Zorko B. (2000). Optimisation of floating matrix tablets and evaluation of their gastric residence time. Int. J. Pharm..

[23] Whitehead L., Fell J.T., Collett J.H., Sharma H.L., Smith A.-M. (1998). Floating dosage forms: An in vivo study demonstrating prolonged gastric retention. J. Control. Release.

[24] Hilton A.K., Deasy P.B. (1992). In vitro and in vivo evaluation of an oral sustained-release floating dosage form of amoxycillin trihydrate. Int. J. Pharm..

[25] He W., Li Y., Zhang R., Wu Z., Yin L. (2014). Gastro-floating bilayer tablets for the sustained release of metformin and immediate release of pioglitazone: Preparation and *in vitro/in vivo* evaluation. Int. J. Pharm..

[26] Hajare A.A., Patil V.A. (2012). Formulation and characterization of metformin hydrochloride floating tablets. Asian J. Pharm. Sci..

[27] Raju D.B., Sreenivas R., Varma M.M. (2010). Formulation and evaluation of floating drug delivery system of metformin hydrochloride. J. Chem. Pharm..

[28] Rajab M., Jouma M., Neubert R.H.H., Dittgen M. (2014). Influence of water-soluble polymers on the in vitro performance of floating mucoadhesive tablets containing metformin. Drug Dev. Ind. Pharm..

[29] Senjoti F.G., Mahmood S., Jaffri J.M., Mandal U.K. (2016). Design and *in-vitro* evaluation of sustained release floating tablets of metformin HCl based on effervescence and swelling. Iran. J. Pharm. Sci..

[30] Kumar R. (2010). Development and in vitro evaluation of sustained release floating matrix tablets of metformin hydrochloride. Int. J. Pharm. Sci..

[31] Rajab M., Jouma M., Neubert R.H., Dittgen M. (2010). Optimization of a metformin effervescent floating tablet containing hydroxypropylmethylcellulose and stearic acid. Pharmazie.

[32] Djebbar M., Chaffai N., Bouchal F., Aouf N. (2019). Effervescent floating tablets of metformin HCl developed by melt granulation. Part I: Effect of hydrophilic polymer on biopharmaceutical properties. GSCBPS.

[33] Djebbar M., Chaffai N., Bouchal F. (2020). Development of floating tablets of metformin HCl by thermoplastic granulation. Part II: In vitro evaluation of the combined effect of acacia gum/HPMC on Biopharmaceutical performances. Adv. Pharm. Bull..

[34] Chandira R.M., Arafath A.A.M.Y., Bhowmik D., Jayakar1 B., Kumar K.P.S. (2012). Formulation and evaluation of bilayered floating tablets of metformin hydrochloride. Pharma Innovation.

[35] Boldhane S.P., Kuchekar B.S. (2009). Gastroretentive drug delivery of metformin hydrochloride: Formulation and in vitro evaluation using 3^2^ full factorial design. Curr. Drug Deliv..

[36] Patel M.B., Shaikh F., Patel V., Surti N.I. (2017). Application of simplex centroid design in formulation and optimization of floating matrix tablets of metformin. J. Appl. Pharm. Sci..

[37] Thapa P., Jeong S.H. (2018). Effects of formulation and process variables on gastroretentive floating tablets with a high-dose soluble drug and experimental design approach. Pharmaceutics.

[38] Eisenächer F., Garbacz G., Mäder K. (2014). Physiological relevant in vitro evaluation of polymer coats for gastroretentive floating tablets. Eur. J. Pharm. Biopharm..

[39] Huh H.W., Na Y.-G., Kang H.C., Kim M., Han M., Pharm T.M.A., Lee H., Baek J.-S., Lee H.-K., Cho C.W. (2021). Novel self-floating tablet for enhanced oral bioavailability of metformin based on cellulose. Int. J. Pharm..

[40] Oh T.-O., Kim J.-Y., Ha J.-M., Chi S.-C., Rhee Y.-S., Park C.-W., Park E.-S. (2013). Preparation of highly porous gastroretentive metformin tablets using a sublimation method. Eur. J. Pharm. Sci..

[41] Pandit V., Pai R.S., Yadav V., Devi K., Surekha B.B., Inamdar M.N., Suresh S. (2013). Pharmacokinetic and pharmacodynamics evaluation of floating microspheres of metformin hydrochloride. Drug Dev. Ind. Pharm..

[42] Jain S.K., Gupta A. (2009). Development of Gelucire 43/01 beads of metformin hydrochloride for floating delivery. AAPS PharmSciTech.

[43] Xu G., Groves M.J. (2001). Effect of FITC-dextran molecular weight on its release from floating cetyl alcohol and HPMC tablets. J. Pharm. Pharmacol..

[44] Ziaee A., Albadarin A.B., Padrela L., Femmer T., O’Reilly E., Walker G. (2019). Spray drying of pharmaceuticals and biopharmaceutical s: Critical parameters and experimental process optimization approaches. Eur. J. Pharm. Sci..

[45] Vehring R. (2008). Pharmaceutical particle engineering via spray drying. Pharm. Res..

[46] Jacyna J., Kordalewska M., Markuszewski M.J. (2019). Design of Experiments in metabolomics-related studies: An overview. J. Pharm. Biomed. Anal..

[47] Maniyar M.G., Kokare C.R. (2019). Formulation and evaluation of spray dried liposomes of lopinavir for topical application. J. Pharm. Investig..

[48] Sander C., Madsen K.D., Hyrup B., Nielsen H.M., Rantanen J., Jacobsen J. (2013). Characterization of spray dried bioadhesive metformin microparticles for oromucosal administration. Eur. J. Pharm. Biopharam..

[49] Pandey K.U., Joshi A., Dalvi S.V. (2021). Evaluating the efcacy of diferent curcumin polymorphs in transdermal drug delivery. J. Pharm. Investig..

[50] Lee E.H., Boerrigter S.X.M., Byrn S.R. (2010). Epitaxy of a structurally related compound on the (100) faces of flufenamic acid form I and III single crystals. Cryst. Growth Des..

[51] JimCnez-Castellanos M.R., Zia H., Rhodes C.T. (1994). Design and testing in vitro of a bioadhesive and floating drug delivery system for oral application. Int. J. Pharm..

[52] Zhang L., Mao S. (2017). Application of quality by design in the current drug development. Asian J. Pharm. Sci..

[53] Lourenço V., Lochmann D., Reich G., Menezes J.C., Herdling T., Schewitz J. (2012). A quality by design study applied to an industrial pharmaceutical fluid bed granulation. Eur. J. Pharm. Biopharam..

[54] Efron B. (1981). Nonparametric estimates of standard error: The jackknife, the bootstrap and other methods. Biometrika.

[55] Efentakis M., Vlachou M. (2000). Evaluation of high molecular weight poly (oxyethylene) (polyox) polymer: Studies of flow properties and release rates of furosemide and captopril from controlled-release hard gelatin capsules. Pharm. Dev. Technol..

[56] Yin X., Li H., Guo Z., Wu L., Chen F., Matas M., Shao Q., Xiao T., York P., He Y. (2013). Quantification of swelling and erosion in the controlled release of a poorly water-soluble drug using synchrotron x-ray computed microtomography. AAPS J..

[57] Al-Zoubi N., Odeh F., Nikolakakis I. (2017). Co-spray drying of metformin hydrochloride with polymers to improve compaction behavior. Powder Technol..

[58] Cesur S., Cam M.E., Sayın F.S., Su S., Gunduz O. (2020). Controlled Release of Metformin Loaded Polyvinyl Alcohol (Pva) Microbubble/Nanoparticles Using Microfluidic Device for the Treatment of Type 2 Diabetes Mellitus.

[59] Tiwari R., Gupta A., Joshi M., Tiwari G. (2014). Bilayer tablet formulation of metformin HCl and acarbose: A novel approach to control diabetes. PDA J. Pharm. Sci. Technol..

[60] Benmessaoud I., Koutchoukali O., Bouhelassa M., Nouar A., Veesler S. (2016). Solvent screening and crystal habit of metformin hydrochloride. J. Cryst. Growth.

[61] Eriksson L., Johansson E., Kettaneh-Wood N., Wikström C., Wold S. (2008). Design of Experiments: Principles and Applications.

[62] van der Voet H. (1994). Comparing the predictive accuracy of models using a simple randomization test. Chemom. Intell. Lab. Syst..

[63] DiFoggio R. (1995). Examination of some misconceptions about near-infrared analysis. Appl. Spectrosc..

[64] Korsmeyer R.W., Gurny R., Doelker E., Buri P., Peppas N.A. (1983). Mechanisms of solute release from porous hydrophilic polymers. Int. J. Pharm..

[65] Shah V.P., Tsong Y., Sathe P., Liu J.-P. (1998). In vitro dissolution profile comparison-Statistics and analysis of the similarity factor, f_2_. Pharm. Res..

[66] Mendyk A., Pacławski A., Szlek J., Jachowicz R. (2013). PhEq_bootstrap: Open-source software for the simulation of f_2_ distribution in cases of large variability in dissolution profiles. Dissolution Technol..

[67] Noce L., Gwaza L., Mangas-Sanjuan V., Garcia-Arieta A. (2020). Comparison of free software platforms for the calculation of the 90% confidence interval of f_2_ similarity factor by bootstrap analysis. Eur. J. Pharm. Sci..

[68] Siepmann J., Peppas N.A. (2012). Modeling of drug release from delivery systems based on hydroxypropyl methylcellulose (HPMC). Adv. Drug Deliv. Rev..

[69] Iglesias N., Galbis E., Romero-Azogil L., Benito E., Lucas R., García-Martín M.G., de-Paz M.-V. (2020). In-depth study into polymeric materials in low-density gastroretentive formulations. Pharmaceutics.

[70] Lamoudi L., Chaumeil J.C., Daoud K. (2016). Swelling, erosion and drug release characteristics of sodium diclofenac from heterogeneous matrix tablets. J. Drug Deliv. Sci. Technol..

[71] Prinderre P., Sauzet C., Fuxen C. (2011). Advances in gastro retentive drug-delivery systems. Expert Opin. Drug Deliv..

[72] Mandal U.K., Chatterjee B., Senjoti F.G. (2016). Gastro-retentive drug delivery systems and their in vivo success: A recent update. Asian J. Pharm. Sci..

[73] Li C.L., Martini L.G., Ford J.L., Roberts M. (2005). The use of hypromellose in oral drug. J. Pharm. Pharmacol..

