# Supplementary material for: DoE-Based Design of a Simple but Efficient Preparation Method for a Non-Effervescent Gastro-Retentive Floating Tablet Containing Metformin HCl
Source: Pharmaceutics. 2021 Aug 8;13(8):1225. doi: 10.3390/pharmaceutics13081225 (PMC8399106; doi:10.3390/pharmaceutics13081225)
Supplement: Supplementary file 1 [file pharmaceutics-13-01225-s001.zip › pharmaceutics-1331973-supplementary.pdf]

pharmaceutics

Supplementary Materials: DoE-Based Design of a Simple but Efficient Preparation Method for a Non-Effervescent Gastro-Retentive Floating Tablet Containing Metformin HCl

Byungsuk Kim, Youngjoo Byun and Eun Hee Lee

Table S1. The full factorial design for a screening step and dissolution profiles (mean ± SD, *n* = 12), and information on similarity factors, floating lag time and floating retention time of non-EFTs.

Run order	X variable (Factor)		Y variable (Response)			Sample mean (f_2)	FLT (s)	FRT (h)
	(A)Cetyl alcohol (mg)	(B)HPMC (%)	(R1)Mean diss. at 60min (%)	(R2)Mean diss. at 240min (%)	(R3)Mean diss. at 480min (%)			
F1	50	5	53.0±3.5	94.4±2.6	98.9±1.6	29.35	-	Not
F2	50	5	51.1±3.2	92.5±3.0	98.7±1.0	30.56	-	Not
F3	250	5	44.1±0.7	78.5±1.4	91.0±1.3	41.90	< 3	> 24
F4	250	5	43.4±0.6	77.7±1.0	90.4±1.8	42.92	< 3	> 24
F5	50	25	41.6±2.9	74.0±3.2	94.0±2.0	44.67	< 3	> 24
F6	50	25	41.0±1.2	73.4±1.8	93.3±2.9	45.77	< 3	> 24
F7	250	25	30.9±0.9	58.1±1.4	75.3±1.7	69.38	< 3	> 24
F8	250	25	31.4±0.6	58.6±1.0	76.0±1.5	71.46	< 3	> 24
F9	150	15	38.6±1.0	74.1±1.2	92.1±1.0	47.26	< 3	> 24
F10	150	15	37.4±0.4	73.4±1.4	92.0±1.6	48.50	< 3	> 24

Table S2. The response surface methodology for optimization step and dissolution profiles (mean ±SD, n=12), and information on similarity factors, floating lag time and floating retention time of non-EFTs.

Run order	X variable (Factor)		Y variable (Response)			Sample mean (f_2)	FLT (s)	FRT (h)
	(A) Cetyl alcohol (mg)	(B) HPMC (%)	(R1) Mean diss. at 60min (%)	(R2) Mean diss. at 240min (%)	(R3) Mean diss. at 480min (%)			
M1	150	15	39.6±0.4	73.5±0.5	91.8±0.5	47.36	< 3	> 24
M2	250	15	37.1±1.5	67.9±2.2	86.4±2.2	58.15	< 3	> 24
M3	150	25	34.1±0.6	64.8±1.5	84.3±2.1	69.08	< 3	> 24
M4	250	25	30.2±1.1	58.2±2.3	77.1±3.3	75.47	< 3	> 24
M5	150	20	37.0±0.6	69.0±1.5	88.5±2.0	55.41	< 3	> 24
M6	250	20	33.1±0.8	62.9±1.2	83.2±1.9	76.76	< 3	> 24
M7	200	15	38.1±1.1	68.9±1.0	86.0±0.8	56.15	< 3	> 24
M8	200	25	32.8±0.7	62.3±1.1	79.4±1.7	77.88	< 3	> 24
M9	200	20	34.5±0.7	64.8±1.0	80.1±1.6	69.32	< 3	> 24
M10	200	20	34.1±0.6	63.8±1.1	79.6±1.2	71.74	< 3	> 24

Table S3. Comparison of dissolution profiles (mean ± SD, *n* = 12) for the external validation set, model prediction accuracy and bootstrap analysis.

Code	Cetyl alcohol (mg)	HPMC (%)	Cont.	60 min (%)	240 min (%)	480 min (%)	Sample mean (f_2)	Bootstrap analysis (500)			
								f_2	E(f_2)	PI	Bcα
			Gluco-phage XR	29.4 ± 0.9	60.4 ± 1.2	81.6 ± 1.3	-	-	-	-	-
E1	150	5	True mean	43.9 ± 0.7	82.3 ± 2.6	97.1 ± 1.4	37.68	37.69	37.67	(36.88, 38.49)	(36.84, 38.44)
			Linear Pred. mean	45.6	81.2	98.3	37.18				
			Quad. Pred. mean	45.6	81.2	99.9	36.58				
E2	150	10	True mean	40.9 ± 0.6	77.1 ± 0.5	94.2 ± 0.3	42.98	42.98	42.95	(42.29, 43.62)	(42.35, 43.67)
			Linear Pred. mean	42.7	77.0	94.4	42.13				
			Quad. Pred. mean	42.7	77.0	96.0	41.39				
E3	200	5	True mean	43.3 ± 0.4	79.4 ± 0.8	95.4 ± 0.5	40.09	40.06	40.04	(39.47, 40.69)	(39.50, 40.74)
			Linear Pred. mean	43.9	78.1	95.3	40.58				
			Quad. Pred. mean	43.9	78.1	93.0	41.50				
E4	200	10	True mean	40.2 ± 0.2	73.4 ± 0.9	90.7 ± 1.3	47.68	47.73	47.64	(46.79, 48.53)	(46.91, 48.75)
			Linear Pred. mean	41.0	73.9	91.4	46.45				
			Quad. Pred. mean	41.0	73.9	89.1	47.55				
E5	250	5	True mean	44.1 ± 0.6	77.1 ± 1.1	91.9 ± 0.8	42.40	42.45	42.43	(41.69, 43.18)	(41.70, 43.19)

			Linear Pred. mean	42.2	75.1	92.4	44.46				
			Quad. Pred. mean	42.2	75.1	93.9	43.74				
E6	250	10	True mean	39.6 ± 0.9	72.0 ± 1.1	87.9 ± 2.0	50.70	50.81	50.64	(49.41, 51.73)	(49.43, 51.79)
			Linear Pred. mean	39.3	70.9	88.5	51.60				
			Quad. Pred. mean	39.3	70.9	90.0	50.68				
	Linear model		RMSEP	1.34	1.18	0.66					
	Quadratic model		RMSEP	1.34	1.18	2.17					

PI: The percentile confidence interval; Bcα: The bias corrected and accelerated confidence interval, RMSEP: The root mean squared error of prediction.

Table S4. Comparison of the dissolution profiles for the optimized formulations predicted using the RSM with the experimentally obtained dissolution profiles.

Code	Cetyl alcohol (mg)	HPMC (%)	Mean dissolution profile	60min (%)	240min (%)	480min (%)	Sample mean (f_2)
P1	150	16	Predicted	39.14	71.99	89.74	50.04
			Experimentally obtained	39.1 ± 0.7	72.2 ± 0.9	89.7 ± 1.5	49.85
P2		17	Predicted	38.55	71.16	88.96	51.68
			Experimentally obtained	38.1 ± 0.3	70.7 ± 0.3	89.0 ± 0.9	52.41
P3		18	Predicted	37.96	70.33	88.18	53.44
			Experimentally obtained	37.2 ± 0.4	70.3 ± 0.4	88.5 ± 0.4	53.80
P4		19	Predicted	37.37	69.49	87.40	55.36
			Experimentally obtained	37.3±0.5	69.6±0.9	88.4±1.1	54.63

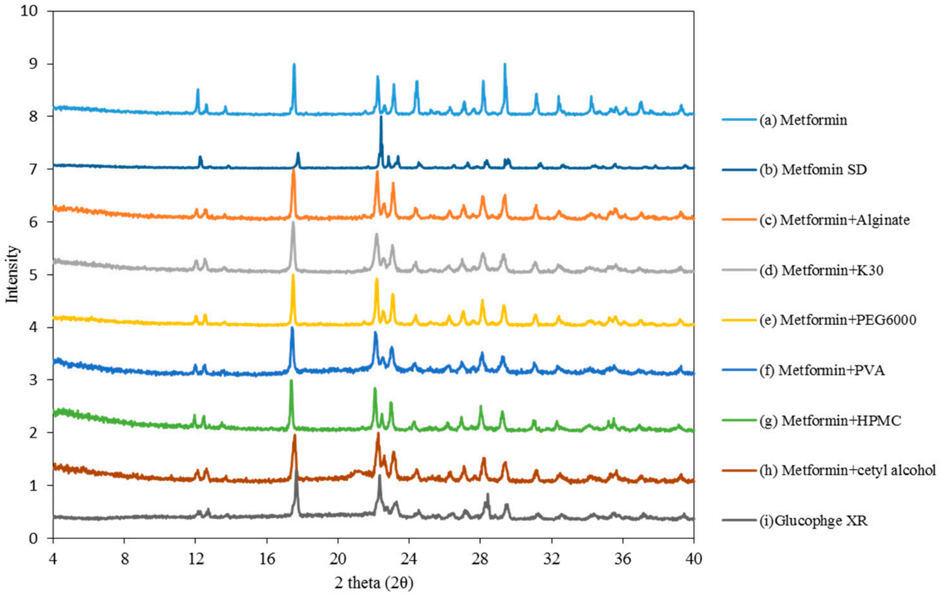

Figure S1. PXRD patterns for crystalline metformin HCl, co-spray dried solid dispersions, and Glucophage XR.

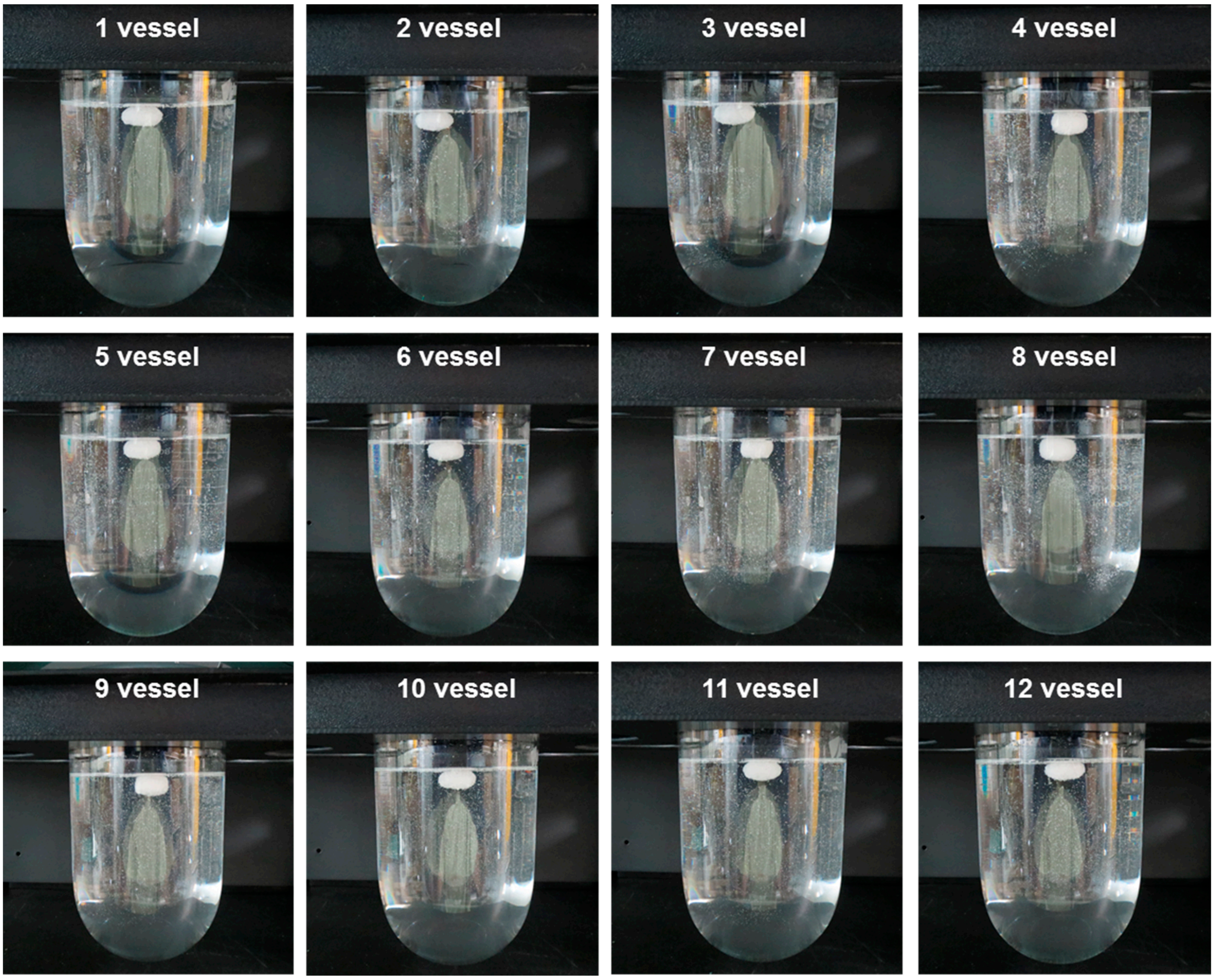

Figure S2. Images of non-EFTs in the vessels after the 24-h dissolution test (*n* = 12).

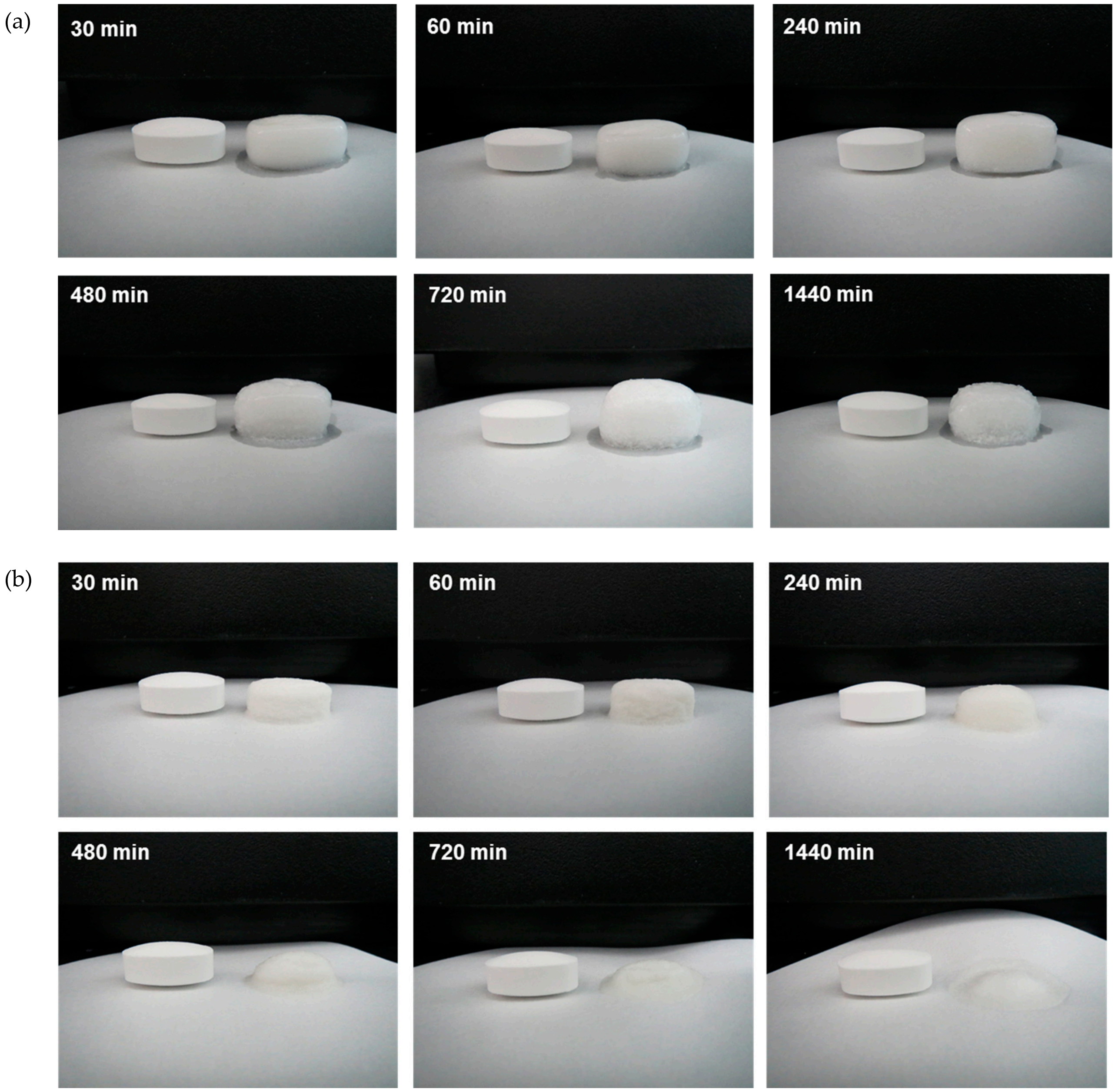

Figure S3. Images of non-EFTs during (**a**) swelling and (**b**) erosion tests over time. Non-EFTs were taken out from the vessels at predetermined times during the dissolution test.
